# What Price Screening?—I

**Published:** 1970-04

**Authors:** A. J. Rowland

**Affiliations:** Principal Medical Officer (Epidemiology), Bristol Department of Health and Social Services


### What Price Screening? — I

### -- a look at the prospects in Bristol by

**A. J. Rowland, M.B., Ch.B., D.P.H.**

Principal Medical Officer (Epidemiology), Bristol Department of Health and Social Services.

Although the battle is by no means over, acute infectious disease has retreated before the advance of modern medicine. The importance of the chronic and degenerative diseases has increased accordingly. The roots of this type of ill health run deep, for the clinical syndrome is the end point of a process which may have been going on for years. Bothwell (1965) has compared its advance to progression through the successive parts of a spectrum which represents not only the pattern of disease in a community, but also its progression in the individual in terms of time. In the first part of Bothwell's spectrum we find those who are free of disease. These will be, in the main, the relatively young, although even amongst these there will be some who have a genetic predisposition to the development of disease in later life. The next part represents the group who are at special risk, perhaps because of the type of environment in which they live. Some of these, in time, pass through the later stages of early biochemical or physical deviations from normal, and early asymptomatic disease. Finally symptoms of ill health begin to occur. When these patients become aware of their symptoms and consult their doctors, they have reached the 'surrender point'. Although the existence of disease is now recognised, and reparative action—treatment—can be taken, the opportunity for prevention has been lost.

Primary prevention consists of removing the fundamental cause of a disease, so preventing its inception. Secondary prevention entails action to disturb the sequence of pathogenesis at an early stage, so that further development is avoided, or at least markedly delayed. These are the only categories of true prevention. So-called tertiary prevention aims only at preventing the further deterioration of an already established condition.

In a few instances it is already possible to aim at primary prevention, although it may still be very difficult to put knowledge into effect. A typical example lies in the possibility of reducing the number of cases of carcinoma of the bronchus by doing away with cigarette smoking (Doll, 1967). Where dangerous substances are used in industry, searches for less harmful substitutes will lead to effective primary prevention, as in the rubber industry, where the development of plastic substitutes for the rubber used for insulation in the cable industry has removed the risk of exposure to carcinogenic anti-oxidants. We do not often have enough knowledge to enable us to undertake primary prevention, but we know how to treat and control many conditions, and it is reasonable to think that the earlier application of treatment, in the pre-symptomatic phase, would achieve the goal of secondary prevention. If this is to be done, it is necessary that an active search be undertaken among apparently well people for those who are in the pre-symptomatic stage. Immediately, several questions demand an answer. Are we able to identify such persons by means of a suitable test? If we are able to identify them, are we able to take effective preventive action? How complex will the sorting process be? How long will it take? What will be the long term gain in terms of disease prevention? It is in the search for the answers to these and similar questions that the nucleus of the present dilemma about screening lies.

Screening for developmental defects in young children has for many years been an important part of the local authority's child welfare work, and has included screening for congenital defects such as dislocation of the hip or occult congenital heart disease, impairment of hearing, or vision, or delay in maturation. Infants are screened for phenylketonuria, either by the phenistix, or, more recently, by the Guthrie, Scriver, or similar tests.

In the adult population, screening for tuberculosis and other pulmonary conditions by means of mass radiography has been going on for many years, and the search among the apparently well for cervical cancer or its precursor, carcinoma in situ, has now become widely established. In Bristol, cervical cytology has been available since 1961, and during 1968 some 9,000 smears were taken—6,083 by local authority doctors in clinics or in a large factory, and 2,873 by general practitioners in their surgeries under N.H.S. arrangements. In addition an unknown number of smears will have been taken on 'private' patients. Even so, these figures represent only a small proportion of those women who are eligible for the test; Johnson (1968) showed for the Bristol clinical area, that during the previous year the smears received represented only

### TABLE 1. ESTIMATED RESULTS OF APPLYING CERTAIN SCREENING TESTS TO THE POPULATION OF BRISTOL

| Condition | Population to be Screened | | Screening Tests Used | | Persons Positive to Test | | Positive to Further Investigation | | Refs. |
|---|---|---|---|---|---|---|---|---|---|
| | Description | Number* | Test | Criteria | Rate | * Number† | Rate | * Number† | |
| Carcinoma | | | | | | | | | |
| —Lung | Men over 40 | 81,710 | Chest X-ray | — | 0.9/1000 | 49 | — | — | 18, 3 |
| Breast | Women 40-64 | 71,970 | Clinical Exam'n & Mammography | — | 2.0% | 960 | 2.3/1000 | 110 | 13, 34 |
| —Cervix (in situ) | | | | | Age specific rates | 504 | Age specific rates | 398 | 10 |
| —Cervix (occult invasive) | Women over 25 | 129,340 | Cervical Cytology | — | 1.3/1000 | 112 | — | — | 19 |
| Anaemia | Non-Pregnant Women 20-74 | 122,230 | Haemoglobin Estimation (E.E.L.) | Hb. less than 10 G % | 3.4% | 2,770 | — | — | 9 |
| Bacteriuria | Non-Pregnant Women 20-64 | 100,150 | "Dip-Spoon" on M.S.U. | Over 100,000 organisms per Ml. | 7.1% | 4,740 | — | — | 37 |
| | Pregnant Women | 19,000 | | | 6.0% | 76 | — | — | 20, 21 |
| Diabetes | Persons aged 20-74 | 282,840 | Blood Sugar 2 hours after 50 G glucose | 120 mg per 100 m. or more | 4.5% | 6,684 | 1.4% | 2,080 | 5, 6, 8 23, 24, 35 |
| Glaucoma | Persons aged 40-74 | 175,850 | Schiotz Tonometry | Tension over 25 mm. $H_2O$ | 4.0% | 4,688 | 1.0% | 1,172 | 2, 11, 16, 25, 27, 30, 31 |

* Over three years.

† Assuming 66% acceptance.

about 12% of the eligible population. This is clearly insufficient to make any measurable impression on the occurrence of carcinoma of the cervix in the area. The Mass Radiography Service provided by the Regional Hospital Board examines some twenty to thirty thousand persons a year in the Bristol area. These are to some extent a selected group; they are partly referrals from general practitioners and partly persons thought to be at special risk—for instance, contacts of known cases of tuberculosis. In 1967 (35,767 examinees) 78 new cases of carcinoma of the bronchus and 24 new cases of tuberculosis were detected, the corresponding figures for 1968 (25,856 examinees) were 56 and 20 respectively.

At present, such efforts are carried out at various points in the city, by various bodies, and in a relatively unco-ordinated fashion. There are other screening tests available. Would it not be more efficient and economical to provide multiphasic screening clinics at which all available tests could be carried out at one time?

If, in the present state of knowledge, a massive screening programme were to be mounted in Bristol, how much work would be generated, and how much disease would be uncovered?

### ORGANISATION AND PROBABLE RESULTS

It would clearly be wise to restrict screening activities to those groups in the population most likely to benefit. The search for phenylketonuria must be aimed at the newborn, the search for carcinoma of the cervix at the woman who is most likely to be in the non-invasive or early invasive stage of development of the disease. Thus, as far as adult screening is concerned, it might be thought that the age group 20 to 74 should be the widest at which screening should be aimed, and that within this wide range, certain sub-groups should be defined for whom some techniques are particularly suitable. The search for early breast cancer by means of mammography, will be most productive of early cases if women aged over 40 are screened, and nullipara might be considered to be at greater risk, so that particular efforts should be directed at persuading these to attend for examination. Persons with a family history of glaucoma are more likely to develop the condition themselves, so that such a history should be given due weight when selecting candidates for tests although, in general, screening the over forties will result in the maximum benefit from glaucoma screening. Factors such as age, social class, parity, and marital status should be taken into consideration when deciding on the choice of groups eligible for cervical cytology programmes. Bacteriuria is more likely to occur in females, and the presence or absence of pregnancy may well be related to the significance of the finding.

In an exercise analogous to that of Last (1963) the probable pick-up rates can be applied to the relevant population of the city, and the potential results estimated. Table 1 represents the results of such an exercise. The tests have been applied to selected groups of the population, in which the finding rates might make the exercise worth-while. It is apparent that not all those eligible for the tests will attend, and the empirical assumption has been made that about two-thirds will in fact be screened. The population used is that based on the 1966 10% sample census. As a result of the initial screening test a group of individuals is produced who have failed the test and need to be more thoroughly investigated. The false positives have to be eliminated by more stringent second tier tests. The false negatives, having, as it were, fallen through the net, are lost to further action. The proportion of false negatives can be reduced by adjusting the criterion of the screening test, but this almost inevitably reduces its sensitivity so that a larger number of false positives is produced. The diagnostic examinations are much more time consuming than the screening tests, and require specialised expertise for successful completion. Before any screening programme can be undertaken, it is necessary to ensure that the extra work load generated can be adequately absorbed by the available clinical and laboratory facilities. In addition, facilities are required for the continued supervision of borderline cases which fail to fall neatly into either positive or negative categories.

It is plain that the provision of screening facilities on a general mass basis for a city the size of Bristol would be a challenging task. There are in this city some 283,000 persons between the ages of 20 and 74. Assuming that two-thirds of these would make use of any facilities provided, and that the intention would be to achieve a three yearly turnover to allow for re-examination at regular intervals, it would be necessary to provide for the examination of nearly 63,000 persons per annum. Each attender would take only those tests for which he or she was eligible, thus while all would take the test for diabetes, only about 24,000 would be eligible for the examination for breast cancer in each year. The demand could be met by twelve screening centres deployed around the city. These could, for instance, be parts of Health Centres, and could work on an appointments basis, examining 22 persons per day.

### COST

Running costs would obviously be related to the range of examinations offered, and the number of staff employed. The estimates which follow are based on the concept of a centre offering the tests shown in table 2, and staffed by a doctor, a clerk, a typist, two nurses (S.R.N.) and two technicians.

### Table 2
**Tests which might be available at a screening clinic**

| | |
|---|---|
| Audiometry | Mammography |
| Bacteriuria | Proteinuria |
| Blood pressure | Serum cholesterol |
| Blood sugar | Tonometry |
| Cervical cytology | Vision |
| Chest X-ray | Visual fields |
| E.C.G. | Peak flow |
| Glycosuria | Mental health (questionnaire) |
| Haemoglobin | |

The provision of equipment for the various tests would cost in the region of £300 per centre. This does not of course include the cost of X-ray apparatus; or the provision of analytical equipment. Mammography and chest X-rays would be particularly important for the over forty age group, and it would seem reasonable to select three or four centres at which these could attend and equip only these centres with X-ray machines. Analysis would best be carried out at one central laboratory. The largest proportion of running costs would be contributed by wages and salaries, which would cost about £11,000 per annum for each centre. Material and laboratory time for the tests would amount to another £2,800. Thus at present day prices, each centre would cost in the region of £13,800 per annum for staffing, administration and test costs, and to this would be added other costs, such as lighting, heating, maintenance, etc. The annual cost to the city for all the centres would therefore be in the region of at least £165,000 per annum—about £2.12.0 per individual screened.

Each year, about 320 breast cancer suspects would require investigation by the surgeons, and approximately 37 would eventually need a mastectomy. The gynaecologists would have to investigate about 160 women, and carry out 130 hysterectomies. In the first sweep through the population about 40 early invasive carcinomas of the cervix would come to light annually although after the first few years, assuming that a substantial proportion of women were being reached, it might be expected that the number of invasive cancers would fall. Heavy loads of work would be generated for the diabetic clinics, with the prospect of some 2,230 glucose tolerance tests a year, and ophthalmic clinics, with the investigation of about 1,560 ocular hypertensives. In these two specialties also, there would be a large number of borderliners who would require observation for a prolonged period.

It is quite apparent that such demands would easily swamp existing facilities. There would be a need to provide for at least some of the second tier tests to be carried out as an extension of the work of the screening clinics. This would call for extra specially trained staff to carry out this work. For instance, tonography and ophthalmoscopy could be carried out on those who failed the preliminary screening tests for glaucoma at a borderline level, and similarly additional tests such as full glucose tolerance tests could be carried out on 'borderline diabetics.' If such second tier tests were done by doctors associated with the initial screening clinics, and only the certain failures from these tests went forward to the hospital clinics, a great deal of the potential load on the consultant clinics would be diverted.

The efficient handling of the records of such a large part of the population of the city, with provision for the accumulation of the results for each individual, and recall for second tier examinations, or simply for further screening sessions as the years passed, would require the use of a computer.

No programme of this sort could possibly be contemplated without a clear indication that the return, however it might be measured, would be commensurate. We need to know what would really be achieved in terms of the prevention of disease. What would this represent in the future? Less expenditure on the treatment of established disease? Fewer demands on supporting social services? A healthier and more productive population? Such assessments can only be made on the basis of experience with screening projects which are already established.

### PAST EXPERIENCE

There is nothing particularly new about the concept of screening for occult disease. Screening for hookworm, malaria, and other infectious disease was carried on from 1910 in the U.S.A., and a manual of 'suggestions for the conduct of periodical examinations of apparently healthy persons' was issued by the American Medical Association in 1927. Mass radiography techniques were developed from the early 1930's, and mass miniature radiography clinics were introduced by the Ministry of Health in 1943. In many ways mass radiography represented an ideal method of screening, for it was a way of detecting a relatively common disease which was undeniably important. The test used was capable of finding the condition sought in its early stages, before it had become symptomatically apparent to the victim. The disease was capable of treatment which would arrest and even reverse the pathological process, and the test was rapid, easy to apply, and acceptable to the patient. It is not surprising that attempts were made to extend the concept in other directions.

Diabetes detection was attempted in the U.S.A. in the early forties (Gates, 1942; Wilkerson and Krall, 1947). Early attempts in the United Kingdom were often attempted as extensions of the activities of Mass Radiography Units—for example, Burn (1956). Most studies showed that an unknown diabetic existed for each one already recognised. Reid (1960) demonstrated that 19.7% of a group of 352 diabetics had suffered continuous symptoms for over a year before the diagnosis was established. The Bedford study (Sharp, Butterfield and Keen, 1964) has been the largest definitive study of diabetes detection in this country. Four per cent of 25,700 specimens of urine were positive to the clinistix test for glucose, and these persons were asked to undergo glucose tolerance tests. Just under a third of them were found to have diabetes, the criterion being a blood sugar level of 120 mg % or more two hours after a 50 G glucose load. However, in the course of the same study 70 of 543 aglycosurics who were randomly subjected to a glucose tolerance test were found to have abnormalities of glucose tolerance. This threw grave doubts on the reliability of simple urine testing as a screening test, a finding later reinforced by the results of a study by the College of General Practitioners (1962).

The distribution of two hour blood sugar levels is continuous, and there is no definable cut-off point at which one ceases to be a non-diabetic, and becomes a diabetic. Between the certainly normal and the certainly abnormal lies a group some of whom will eventually become frankly diabetic (O'Sullivan and Mahan, 1965). If secondary prevention is our aim, this is the group in which we are intensely interested, yet we do not know how to predict which of its members will become diabetic, still less do we know if some form of treatment will stop this from happening. Keen is currently engaged on a study of the Bedford borderliners which is designed to shed some light on these problems, but at present the value of detecting very early diabetes is not proven, and the pre-diabetic state cannot be recognised with certainty.

This story has been paralleled in other directions. Brav and Kirber's study (1951) of glaucoma detection resulted in the identification of a similar borderline group of ocular hypertensives whose fate was uncertain, and this has been the experience in all subsequent studies. Graham (1966) has expressed considerable doubts about the use of tonometry alone as a screening test, and the combination of tonometry with ophthalmoscopy, or with visual field screening (using rapid methods) has been suggested in the search for a more reliable test. The phenistix test for phenylketonuria was widely applied in the United Kingdom from 1959 onwards. By 1963, the first doubts about its reliability were being expressed (Scott, 1963; Woolf, 1967) and in due course Stephenson and McBean (1967) demonstrated that four out of twelve cases had been missed. In Bristol, although four cases have been found, false negative results occurred in another two. The more reliable Guthrie and similar tests are now coming into use; even so, doubts still remain in some quarters about the effectiveness of the dietary treatment of phenylketonuria (Wilson, 1968). The failure, so far, of the cervical cytology programmes in British Columbia and New Zealand to produce a recognisable reduction in mortality from carcinoma of the cervix has been well documented (Green, 1966), although Fidler and co-workers (1968) have recently reported a significant decline in the incidence of invasive disease. It is possible that the long latent period in the development of invasive disease (Petersen, 1956; Way et al., 1968) coupled with the difficulties in persuading those most at risk of cancer to accept the test, may explain the absence so far of any impact on mortality. Recent investigations of the significance of bacteriuria (Sussman et al., 1969) suggest that the condition is only likely to be worth seeking and treating in the pregnant woman, although current investigations into the condition in school children may produce another group in which the test may be of value. Elwood and colleagues (1967) have thrown considerable doubt on the value of screening for anaemia.

### CONCLUSIONS

Clearly, a stage has been reached when a careful re-appraisal of the usefulness of the concept of screening for pre-symptomatic disease is necessary. Certain questions must be satisfactorily answered with regard to any condition for which it is proposed to undertake screening surveys. They are best derived from Wilson's 'ten points' (Wilson and Jungner, 1968).

1. Is the condition sought an important health problem?
2. Is there an accepted treatment for patients with recognisable disease?
3. Are facilities for diagnosis and treatment available?
4. Is there a recognisable latent or early asymptomatic stage?
5. Is there a suitable test or examination?
6. Is the test acceptable to the population?
7. Is the natural history of the condition, including progression from latent to declared disease adequately understood?
8. Is there an agreed policy on whom to treat as patients?
9. Is the cost of case finding (including diagnosis and treatment of patients diagnosed) economically balanced in relation to possible expenditure on medical care as a whole?
10. Will case finding be a continuous process, rather than a 'once and for all' event?

The application of these questions to the majority of the screening procedures presently available opens up large gaps in current knowledge, and the need is therefore for carefully controlled investigations, related to conditions selected for their potential suitability for screening programmes, with the object of deriving the missing answers. It is particularly important that screening does not become a widespread and accepted technique until it is based on firm, scientifically acceptable foundations. Otherwise there is great danger that it will no longer be ethically acceptable to carry out the very investigations necessary to establish its credibility. Already, it is plain that a controlled trial of the efficacy of treatment of phenylketonuria would be unthinkable, as would a controlled study of the outcome of treating carcinoma in situ. New screening projects must therefore be set up from the outset as specific research ventures, with the intention of fully documenting and following up all cases, whether an abnormality is detected at screening or not. Where abnormalities are detected, controlled trials of the efficacy of treatment—such as that currently being carried out by Keen—are required. These will define both the natural history of the disease from its earliest stage of inception and the effect, if any, of early treatment upon the progress of the pathological process. Such investigations must be undertaken with due consideration for their anxiety-provoking potential, and in full knowledge of the ethical considerations involved. McKeown (1968) has rightly pointed out that when a doctor or public health medical authority takes the initiative in investigating the possibility of illness or disability in persons who have not complained of signs or symptoms "there is then a presumptive undertaking, not merely that abnormality will be identified if it is present, but that those affected will derive benefit from subsequent treatment or care. This commitment is at least implicit, and *except for research* and the protection of public health . . . no one should be expected to submit to the inconvenience of investigation or the anxieties of case finding without the prospect of medical benefit. The obligation exists even when the patient asks to be screened, for his request is then based on the belief that the procedure is of value, and if it is not, it is for medical people to make this known."

The time is ripe, not for great city-wide schemes of mass population screening, but for studies of the effects and benefits, as well as the feasibility, of the method on smaller, well defined populations. These would be research projects, and must be clearly understood to be such. Such projects might be undertaken within a general practice, where a well defined population, listed on an accurate age/sex register, is available. Or they may be undertaken within the context of occupational health schemes—such a study within the Occupational Health Service of the Corporation of Bristol is in the early stages of development. Adequate research is the immediate price of effective and ethically acceptable screening. Unless this is paid in full at the outset, there can be no real progress in this, as in any other, field of medicine. The cost benefit analysis can safely be left to the future, when we may hope to have better evidence upon which to base our estimations.

### REFERENCES

1. **Bothwell, P. W.** (1965) in 'A New Look at Preventive Medicine', Pitman.

2. **Brav, S. S., and Kirber, H. P.** (1951). J. Amer. med. Ass. **147,** 1127.

3. **Brett, G. Z.** (1966) Proc. roy. Soc. Med. **59,** 1208.

4. **Burn, J. L.** (1956) Med. Offr. **96,** 5.

5. **Canelo, C. K.** et al (1949) Calif. Med. **71,** 409.

6. **College of General Practitioners** (1962) Brit. med. J. **1,** 1497.

7. **Doll, R.** (1967) in 'The prevention of cancer; pointers from epidemiology', Oxford University Press.

8. **Donaldson, R. J., and Howell, J. M.** (1965) Brit. med. J. **2,** 1034.

9. **Elwood, P. C.** et al (1967) Brit. med. J. **4,** 714.

10. **Fidler, H. K., Boyes, D. A., and Worth, A. J.** (1968) J. Obstet. Gynaec. Brit. Cwlth. **75,** 392.

11. **Frydman, J. E.** et al. (1966) J. Amer. med. Ass. **198,** 1237.

12. **Gates, E. W.** (1942) Indust. Med. **11,** 387.

13. **Gershon-Cohen, J.** et al. (1967) Radiology **88,** 663.

14. **Graham, P. A.** (1966) Proc. roy. Soc. Med. **59,** 1215.

15. **Green, G. H.** (1966) Amer. J. Obstet. Gynec. **94,** 1009.

16. **Hollows, F. C., and Graham, P. A.** (1966) Brit. J. Ophthal. **50,** 570.

17. **Johnson, D. H.** (1968) Bull. Med. Off. Hlth. (Bristol), No. 1, p. 3.

18. **Johnston, R. N., and Smith, D. N.** (1968) Lancet **2,** 588.

19. **Kaiser, R. F.** et al. (1960) J. nat. Cancer Inst. **25,** 863.

20. **Kass, E. H.** (1960) Arch. intern. Med. **105,** 194.

21. **Kincaid Smith, P.** et al. (1964) Lancet **2,** 61.

22. **Last, J. M.** (1963) Lancet **2,** 28.

23. **McDonald, G. W.** et al. (1963) Publ. Hlth. Rep. (Wash.) **78,** 553.

24. idem (1965) ibid. **80,** 163.

25. **McDonald, J. E., and Johnson, M. O.** (1956) Amer. J. Ophthal. **59,** 875.

26. **McKeown, T.** (1968) in 'Screening in Medical Care', Oxford University Press.

27. **Meyer, W. J.** (1962) Amer. J. publ. Hlth. **52,** 75.

28. **O'Sullivan, J. B., and Mahan, C. M.** (1965) J. Amer. med. Ass. **194,** 587.

29. **Petersen, O.** (1956) Amer. J. Obstet. Gynec. **72,** 1063.

30. **Porter, G. Leroy** (1958) Trans. Amer. Acad. Ophthal. Otolaryng. **62,** 54.

31. **Power, J. L.** (1957) Amer. J. Ophthal. **44,** 696.

32. **Reid, J. J. A.** (1960) Med. Offir. **103,** 325.

33. **Scott, J. G.** (1963) Brit. med. J. **1,** 1164.

34. **Shapiro, S. et al** (1966) J. Amer. med. Ass. **195,** 731.

35. **Sharp, C. L., Butterfield, W. J. H., and Keen, H.** (1964) Proc. roy. Soc. Med. **57,** 193 et seq.

36. **Stephenson, J. B. P., and McBean, M. S.** (1967) Brit. med. J. **3,** 582.

37. **Sussman, M.** et al (1969) Brit. med. J. **1,** 799.

38. **Way, S.** et al (1968) J. Obstet. Gynaec. Brit. Cwlth. **75,** 593.

39. **Wilkerson, H. L. C., and Krall, L. P.** (1947) J. Amer. med. Ass. **135,** 209.

40. **Wilson, J. M. G.** (1968) in 'Screening in Medical Care', Oxford University Press.

41. **Wilson, J. M. G., and Jungner, G.** (1968) Principles and Practice of Screening for Disease. Public Health Papers, No. 34, W.H.O., Geneva.

42. **Woolf, L. J.** (1967) Brit. med. J. **3,** 862.

